# Wide-Field Detected Fourier Transform CARS Microscopy

**DOI:** 10.1038/srep37516

**Published:** 2016-11-24

**Authors:** Alex Soares Duarte, Christoph Schnedermann, Philipp Kukura

**Affiliations:** 1 Department of Chemistry, Physical and Theoretical Chemistry Laboratory, University of Oxford, South Parks Road, Oxford OX1 3QZ, UK

## Abstract

We present a wide-field imaging implementation of Fourier transform coherent anti-Stokes Raman scattering (wide-field detected FT-CARS) microscopy capable of acquiring high-contrast label-free but chemically specific images over the full vibrational ‘fingerprint’ region, suitable for a large field of view. Rapid resonant mechanical scanning of the illumination beam coupled with highly sensitive, camera-based detection of the CARS signal allows for fast and direct hyperspectral wide-field image acquisition, while minimizing sample damage. Intrinsic to FT-CARS microscopy, the ability to control the range of time-delays between pump and probe pulses allows for fine tuning of spectral resolution, bandwidth and imaging speed while maintaining full duty cycle. We outline the basic principles of wide-field detected FT-CARS microscopy and demonstrate how it can be used as a sensitive optical probe for chemically specific Raman imaging.

Optical microscopy has deepened our understanding of the interior design and the intricate working apparatus of cells and biological tissue down to the nanometer scale^1^,^2^,^3^,^4^,^5^. Achieving sufficient imaging contrast typically involves externally introduced labels, which allows for highly specific and sensitive imaging. At the same time, the reliance on labels reduces the dimensionality of the acquired images in terms of the number and identity of detectable species. Label-free and chemically specific imaging could in principle revolutionize our ability to investigate micro- and nanoscopic processes given the potential for multi-species imaging. As a result, numerous label-free imaging modalities have been developed over the past decades. Of these, coherent Raman scattering-based approaches have made remarkable progress, with high-speed imaging now possible not only in specialized environments, but also in tissues and *in vivo*^6^,^7^,^8^,^9^,^10^,^11^,^12^.

Taking advantage of the full potential of Raman imaging for chemical specificity ultimately demands efficient acquisition of high-quality Raman spectra over the chemically informative fingerprint region of the vibrational spectrum (500–1600 cm^−1^)^13^,^14^. Although broadband acquisition is invariably slower compared to acquisition restricted to single vibrational features, it also opens the door to advanced chemometrics such as principle component or multivariate analysis^15^,^16^. The rich information content that can be obtained from such an approach was recently demonstrated in biological tissues using broadband coherent anti-Stokes Raman scattering (b-CARS)^1^,^10^ In b-CARS, the combination of a broadband femtosecond pump/Stokes pulse with a narrow-band picosecond probe pulse (Fig. 1a) generates vibrational coherence over the full vibrational manifold, which is subsequently read out by a second interaction with the pump electric field. The frequency-domain approach to CARS has the advantage of producing broadband spectra in a single laser shot. At the same time, the raw acquired spectra suffer from non-resonant background, which leads to complicated dispersive lineshapes, compared to comparatively narrow Lorentzian lineshapes of a spontaneous Raman spectrum^17^.

Much effort has thus been aimed towards avoiding non-resonant background in CARS microscopy resulting in a number of techniques and algorithms that efficiently resolve the issue^10^,^17^,^18^,^19^. In the frequency-domain, a promising alternative to b-CARS forms femtosecond stimulated Raman microscopy (FSRM), which is less prone to non-resonant background complications^20^. In contrast to b-CARS, FSRM uses narrowband pump pulses in combination with broadband probe pulses to generate Raman gain or loss on top of the recorded probe spectrum resulting in extremely fast acquisition times on par with b-CARS, as recently demonstrated in polymer blends^21^. While FSRM does not struggle with non-resonant background, the temporal overlap of pump and probe pulses often generates broad baseline contributions that can also affect lineshapes^22^.

Background-and baseline free Raman spectra can also be achieved using a time-domain approach based purely on femtosecond pulses (Fig. 1b), termed Fourier transform coherent anti-Stokes Raman scattering (FT-CARS). In FT-CARS, a broadband pump pulse generates a multitude of vibrational coherences via an impulsive Raman process^23^,^24^, which are read out by a time-delayed broadband probe interaction and detected on the high-frequency side of the probe pulse (Fig. 1c). Following Fourier transformation, a background- and baseline-free Raman spectrum is obtained^23^,^24^,^25^,^26^,^27^,^28^,^29^,^30^. A particularly powerful time-domain approach was recently demonstrated based on two synchronized frequency combs, enabling extremely rapid spectral acquisition, but at the expense of drastically lowering the duty cycle^31^. More recently, the combination of a single femtoscond oscillator with a rapid 4f-mirror-scanning delay stage remedied this issue and advanced FT-CARS microscopy to achieve unprecedented spectral acquisition speeds^32^.

Despite the recent improvements in acquisition speeds, all experimental implementations of FT-CARS microscopy presented to date rely on point-by-point-detection, in which a Raman spectrum is recorded separately for each pixel (Fig. 1d). Hyperspectral images are then composed from multiple Raman measurements across the region of interest in the sample either by sample translation or slow beam scanning^27^,^31^,^32^. Here, we combine the advantages of FT-CARS microscopy for delivering background and baseline-free Raman spectra with rapid beam-scanning and wide-field imaging detection typically employed in fluorescence microscopy. The use of a time-domain FT-CARS scheme avoids the need for spectral dispersion required in b-CARS, and enables wide-field imaging. In addition, we can take full advantage of the higher peak intensities of femtosecond over picosecond pulses leading to larger coherent Raman signals per incident average power while minimizing sample damage by rapid two-dimensional beam scanning^33^. In contrast to the conventional point-detection schemes, our approach generates vibrational coherence of the entire sample region of interest time-point by time-point (Fig. 1e) and consequently enables us to use highly-sensitive CCD or sCMOS cameras for maximum detection efficiency. Furthermore, decoupling the imaging resolution from the illumination spot size enables tuning of the excitation intensity through the spot size without loss of imaging resolution. Additional channels, such as second harmonic generation only require the addition of dichroic beamsplitters as in multi-colour fluorescence microscopy, rather than the need for additional point detectors, which must be synchronized with the scanning motion, increasing experimental complexity. We remark that our approach differs considerably from previously reported wide-field CARS microscopy, which does not use tightly focused beams^34^,^35^,^36^. We illustrate the basic concepts of wide-field detected FT-CARS microscopy by examining solvent and bead samples and outline potential future improvements for investigating biological samples.

## Results and Discussion

The implementation of our wide-field detected FT-CARS microscope is illustrated in Fig. 2 (see Methods for more details). An 80 MHz Ti:Sapphire oscillator provides femotsecond pulses at 800 nm which are split into identical pulse copies in a balanced Michelson interferometer with a well-defined inter-pulse separation, Δt. The pulses are then sent to a pair of rapid resonant scanning mirrors, which generate a two-dimensional scanning pattern that is relay-imaged into a home-built transmission microscope resulting in a scanned area of up to 30 × 30 μm^2^ at the sample. The generated CARS signal is subsequently imaged onto an EMCCD camera and excitation light is suppressed using a 750 nm long pass filter in the illumination and a 740 nm short pass filter in the detection path. The dispersion of the entire system is compensated by a pair of chirped mirrors resulting in 18 fs pulses at the sample.

To demonstrate the concepts behind wide-field detected FT-CARS microscopy we begin by imaging a 30 × 30 μm^2^ region of toluene. The non-resonant background generated by pump and probe pulses individually is easily detected and allows for rapid alignment of the correct focal plane for the experiment. Without stepping the time delay between the pump and probe pulses (18 fs, 800 nm, 3.6 mW in each interferometer arm), we observe a scanning pattern with a characteristic harmonic oscillator intensity distribution in two dimensions caused by the resonant scanning motion (Fig. 3a). Additionally, phase drift between the resonant SM for short exposure times (<10 ms) causes Lissajous patterns to appear occasionally.

When stepping the time delay between pump and probe pulses, we resolve clear long-lasting oscillatory modulations on top of the time-independent CARS background (Fig. 3b). The oscillatory amplitude comprises ~4% of the overall detected anti-Stokes signal and exhibits strong coherent artifact contributions at zero time delay when pump and probe pulses are temporally overlapped^31^. To avoid these contributions, we consider only pump-probe delays >200 fs. Fourier transformation following subtraction of the constant background reveals bands at 786, 1003, 1031 and 1210 cm^−1^ in excellent agreement with the characteristic Raman frequencies of toluene (Fig. 3c)^37^.

Our rapid scanning approach enables access to practically simultaneously recorded and spatially-resolved spectral information by performing a Fourier transformation of the time delay dimension. To illustrate this concept, we have selected a single line cut through the center of the scanned region (dashed line Fig. 3a) and computed the Fourier power spectrum at each pixel (Fig. 3d). As previously, we observe the characteristic vibrational modes of toluene with a harmonic intensity variation as a function of the spatially inhomogeneous distribution. It is worth noting, that the Fourier intensity (amplitude) scales quadratically (linearly) with the incident power, allowing for quantitative analysis (Fig. 3d, inset). The data acquisition time for this measurement was 30 s with an average incident power of 3.6 mW in each interferometer arm, amounting to a nominal integration time of 1.8 ms/pixel.

To explore the capabilities of wide-field detected FT-CARS microscopy beyond solvent samples, we investigated a dry mixture of poly(methyl methacrylate) (PMMA) and polystyrene (PS) beads attached to a glass surface. The corresponding Fourier power maps at the most intense fingerprint peaks of both bead samples (PMMA – 810 cm^−1^, PS – 1010 cm^−1^) are shown in Fig. 4a,b 38. Based on these chemical images, we can identify 5 PMMA particles (blue, Fig. 4a) with a diameter of ~0.7 μm as well as 3 larger PS particles (red, Fig. 4b) of ~1.2 μm diameter. We remark that the intrinsic Raman cross section for PS is orders of magnitude larger than for PMMA, resulting in a higher signal-to-noise ratio (Fig. 4b). To judge the overall noise level of our measurement, we have selected an image at 1270 cm^−1^, which is free from Raman signatures of the studied beads (Fig. 4c). Intriguingly, we are still able to retrieve the positions of the beads, based on a higher Fourier intensity, but have lost chemical specificity. We can account for this behavior by considering that the investigated sample consisted of dry spherical 0.5–2 μm beads. By appropriately focusing on the beads and only collecting CARS signals originating from them, we are insensitive to other CARS sources. Consequently, only the bead positions are affected by noise sources such as scanning noise and laser fluctuations. In this sense, the information retrieved in Fig. 4c is analogous to the time-independent CARS image, which is employed for alignment of the focus of the microscope.

The composition image of PMMA and PS maps (Fig. 4d) demonstrates the capability of wide-field detected FT-CARS microscopy to spatially and spectrally resolve chemical signatures including the possibility of z-sectioning. The selected spectra of the studied beads (arrow in Fig. 4a,b) show an excellent signal-to-noise ratio and highlight the potential of our technique for biological imaging. The bead data was derived from a single wide-field detected FT-CARS microscopy trace containing 300 images (15 × 15 μm^2^), which required an acquisition time of 18 seconds at an exposure time of 60 ms leading to an acquisition time of 1.8 ms/pixel. This acquisition speed compares well with current state-of-the-art Raman imaging techniques based in the frequency domain^10^.

## Conclusion

We presented a wide-field time-domain CARS microscope (wide-field detected FT-CARS microscopy) based on rapid spatial beam scanning in combination with sensitive camera imaging. To illustrate the capabilities of wide-field detected FT-CARS microscopy, we investigated toluene and bead samples over a large field of view (up to 30 × 30 μm^2^) and demonstrated ‘fingerprint’ region Raman spectra of high chemical specificity, fast data acquisition (~2 ms/pixel) and excellent suppression of non-resonant background. Beyond these encouraging initial results, there are numerous avenues for future improvements in imaging sensitivity and speed that are exclusively technical in nature and can be achieved with existing, commercially available components.

The currently employed scanning approach introduces time-varying Lissajous patterns (Fig. 3a), which cause additional noise to an otherwise shot-noise limited measurement. This poses a lower limit on the exposure time and is directly linked to the employed scanning amplitude. We found that an integration time of 20 ms and a field of view of 30 × 30 μm^2^ suppresses spatial noise sufficiently for solvents, but higher integration times may have to be chosen for weaker Raman scatters frequently encountered in biological samples. Precise phase control of the SM motion could reduce the exposure time and the spatial noise allowing for faster acquisition. Such an approach, together with using a slightly larger beam focus (~1 μm), will enable larger fields of view. Note that large beam foci can be used here without any deterioration of the spatial resolution because the CARS light is imaged by the collection objective, rather than being focused onto a point detector. The ability to change the incident beam diameter without loss of spatial resolution provides an additional means to fine tune the balance of peak and average power given a fixed, <20 fs pulse duration, which is not available to point scanning approaches without loss in spatial resolution. Confocal sectioning, as in two-photon fluorescence, remains intrinsic to the approach due to the non-linear scaling of the CARS signal with incident power.

Our setup currently employs 18 fs pulses, which is limited by the dispersive optics and filters in the setup. Ti:Sa lasers with <15 fs lasers are readily available, including the unit used here, and have been compressed for high-NA objectives to well below 10 fs^39^. It is worth noting, that the intensity distribution of the retrieved Raman spectrum deviates from the spontaneous Raman distribution and depends on the effective time resolution as well as the employed filters to extinguish excitation light^27^. Reducing the pulse duration to 10 fs would enhance the effective time resolution of the experiment from currently 27 fs to 14 fs. For a 1000 cm^−1^ mode, this would increase the corresponding Fourier intensity 30-fold while a 2000-fold increase is expected for a 1500 cm^−1^ mode^30^. Furthermore, the efficiency of generating vibrational coherences is improved for shorter pulse durations due to the enhanced bandwidth, leading to an additional increase in signal magnitude^30^.

In order to make wide-field detected FT-CARS microscopy applicable to weakly scattering samples, such as biological tissue, photoinduced damage needs to be considered. We found that we could not use average powers above 8 mW (50 pJ) without inducing sample damage, in line with power levels reported elsewhere^10^,^36^. Non-linear effects are the main source of sample damage where the peak-power is the dominant factor, thus, we should be able to employ a 1 GHz laser system, while maintaining comparable average power, leading to an increase in the CARS signal of about an order of magnitude. Alternatively, a tailored multi-pulse single-beam excitation with controlled time delay between consecutive pulses, could be used as reported previously^40^,^41^,^42^. This approach would reduce the exposure time and avoid sample damage, while maintaining the advantages of a time-domain vibrational coherence detection.

There are three intrinsic downsides of wide-field detected FT-CARS microscopy over point-based frequency domain approaches. Firstly, although wide-field imaging provides higher detection efficiency with >90% quantum yield detectors available, time-based confocal sectioning is no longer available meaning that sample scatter can lead to a loss in spatial resolution. Given the success of deep-tissue two photon imaging^43^, however, there is no intrinsic reason why the imaging quality of wide-field detected FT-CARS microscopy should be inferior. Secondly, the use of a short probe pulse only converts a small fraction of the pump-induced vibrational coherence into a measurable signal compared to the more optimized use of ps pulses matching common vibrational dephasing times. This effect, however, is likely to be at least partially compensated by the higher peak intensity afforded by the use of exclusively femtosecond pulses compared to picosecond-femtosecond combinations as employed in b-CARS. Thirdly, due to the long data acquisition time required for hyperspectral time-domain compared to single-mode frequency-domain techniques, Raman imaging of living organisms is likely to be more challenging. Taken together, wide-field detected FT-CARS microscopy should provide a valuable addition to the palette of available Raman-based imaging techniques, with a particular focus on spectral quality and ease of implementation to achieve large field of views.

## Methods

### Setup Design

An ultrafast Ti:Sa oscillator (Vitara-T, Coherent Inc., 80 MHz repetition, 450 mW, 80 nm bandwidth) followed by a balanced Michelson interferometer delivered identical pulse copies with controllable interpulse delay (PI M-230.10, Fig. 2). Fast resonant scanning mirrors (SM, EOPC/SC-30)^36^,^44^ with slightly detuned resonance frequencies (16 kHz and 16.1 kHz) scanned the pulses at the sample over a square region of up to 30 × 30 μm^2^. We highlight that this field of view is not limited by the employed scanning mirrors, but was chosen intentionally to keep additional noise sources (e.g. intermediate Lissajous figures) at a minimum. This could be improved in future implementations by either increasing the focus size or optimized scanning approaches, which are standard in confocal microscopy. Tight focussing was achieved with an oil immersion objective (Plan Achromat, 100x, 1.25 NA). A second oil immersion objective (Plan, 100x, 1.25 NA, Leica) for solvent measurements or an air objective (UMPlanFl, 100x, 0.95 NA, Olympus) for bead samples was used to collect the transmitted light which was subsequently imaged by an achromatic tube lens (F = 250 mm) onto an EMCCD camera (Rolera Thunder, QImaging) with a magnification of 140x for both collection objectives. Inserting a 750 nm long pass filter (FELH0750, Thorlabs) in the illumination path and a 740 nm short pass filter (740CFSP, Omega Optical Inc.) in the imaging path efficiently discriminated the blue-shifted anti-Stokes scattering against the pump/probe background. Third-order compensated chirped mirrors (Layertec) compressed the pulses to 18 fs duration at the focus of the objective as verified by two-photon fluorescence^45^. The beam focus was 450 nm and scanning did not affect the pulse duration more than 0.6 fs along the scanned region. The telecentric imaging system (1:2.5) is based on concave mirrors, which expand the beam, resulting in a sub-micron focus at the sample, while avoiding chromatic aberrations and additional chirp.

### Data Acquisition and Processing

**Figure 1:**
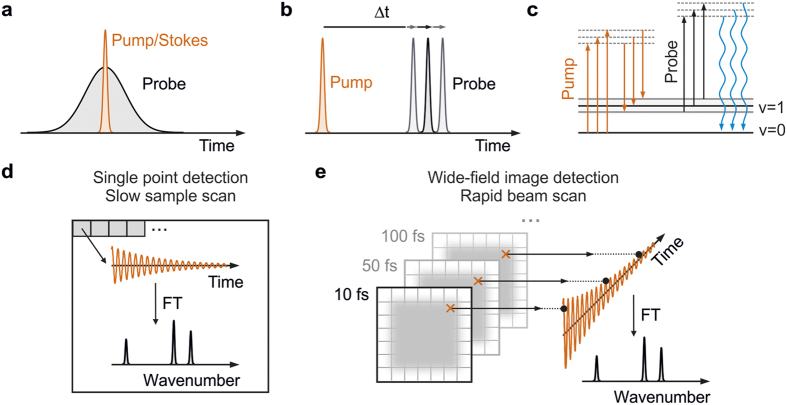
Overview of coherent anti-Stokes Raman scattering (CARS) microscopy. (**a**) Pulse-timing diagram for broadband frequency-domain CARS and (**b**) time-domain CARS. (**c**) Wave-mixing energy ladder diagram describing the generation of blue-shifted CARS photons after three electric field interactions with the sample. Fourier transform CARS microscopy relying on single point (**d**) and wide-field image (**e**) detection. CARS spectra are retrieved after Fourier transformation (FT) of the obtained coherent oscillations.

**Figure 2:**
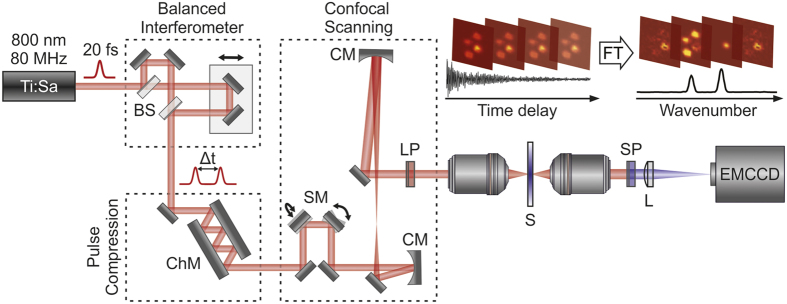
Wide-field detected FT-CARS microscopy setup scheme and concept. Combination of a balanced interferometer, chirped mirror compression and reflective beam scanning delivers 18 fs pulses to the focus of a high NA microscope objective. Complete images are read out for each interpulse time delay before being Fourier transformed to produce a hyperspectral image. BS – beamsplitter, ChM – chirped mirrors, SM – scanning mirrors, LP – long pass filter, SP – short pass filter, S - sample, L - tube lens, CM - curved mirror.

Data acquisition was carried out by recording the vibrational coherence time-point by time-point over the full scanned region (Fig. 2 inset). The CARS image of the sample is recorded as a function of pump-probe time delay resulting in an image series where each pixel represents the local vibrational coherences along the time delay dimension (Fig. 1e). Subsequent Fourier transformation of the image series yields a frequency-resolved vibrational spectrum for each imaged pixel revealing the local sample chemical composition. Thus, we can depict the chemical map of the sample by selecting different Raman modes out of the Fourier transformed dataset.

### Sample preparation

**Figure 3:**
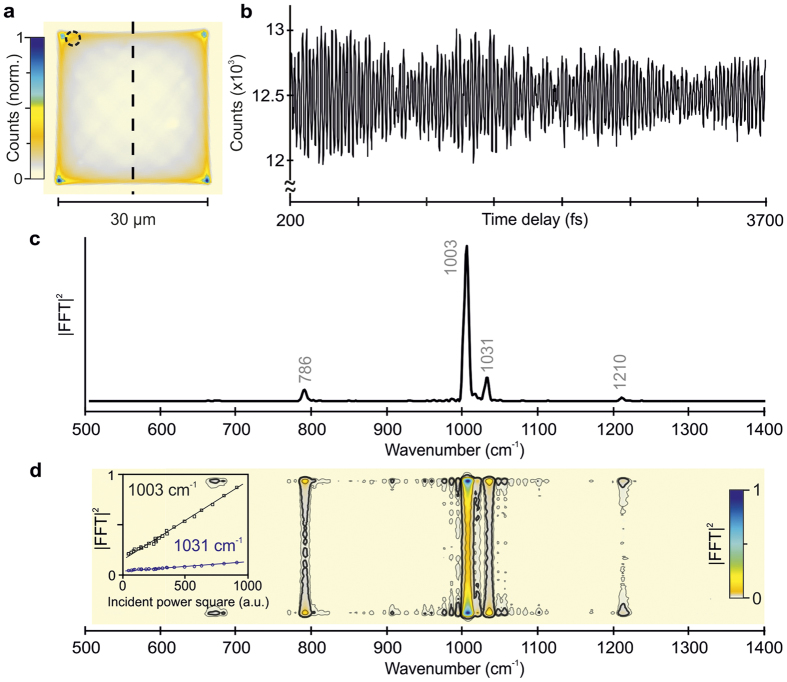
Wide-field detected FT-CARS microscopy on toluene. (**a**) Time-independent coherent anti-Stokes background scattering of the scanning region (30 × 30 μm^2^). The inhomogeneous intensity distribution arises from the harmonic motion of the scanning mirrors. (**b**) Coherent oscillations of toluene recorded for 3.5 ps on a single pixel (dashed circle in **a**). The data was recorded in 30 s with an EMCCD camera integration time of 20 ms in the absence of EM gain. (**c**) Fourier power spectrum of the coherent oscillations in (**a**) showing the characteristic vibrational peaks of toluene. (**d**) Fourier power map along the vertical dashed black line in (**a**). We note, that the Fourier intensity scales quadratically with the incident power (inset).

**Figure 4:**
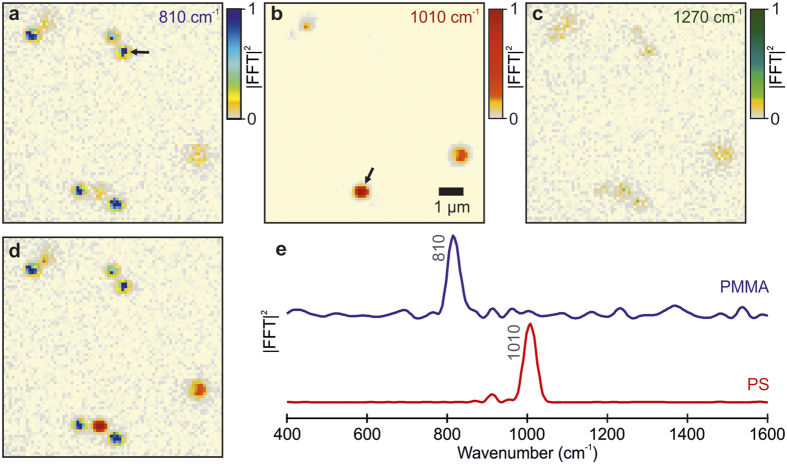
Wide-field detected FT-CARS microscopy applied to a dry mixture of PMMA and PS beads. Normalized Fourier power map (9 × 9 μm^2^) at (**a**) 810 cm^−1^ and (**b**) 1010 cm^−1^ corresponding to the dominant low-frequency modes identifying PMMA and PS beads, respectively. (**c**) Fourier power map at 1270 cm^−1^ characterizing the noise level of the measurement (normalized by the 810 cm^−1^ peak). (**d**) Composite images of (**a**,**b**) illustrating the chemical sensitivity of wide-field detected FT-CARS microscopy. (**e**) Top: Normalized Fourier power spectrum of PMMA bead pointed by the arrow in (**a**) Bottom: normalized Fourier power spectrum of a the PS bead pointed by the arrow in (**b**).

The toluene sample (Fig. 3) was sandwiched between two microscope coverslides resulting in ~140 μm path length. For the bead experiments (Fig. 4), we prepared a dry distribution of poly(methyl methacrylate) (PMMA, 0.5–1.0 μm diameter) and Polystyrene (PS, 0.8–2.0 μm diameter) beads attached to a glass surface^20^,^46^,^47^. The bead samples were prepared by spin coating a drop of the beads mixture (1:1) in methanol solution over a clean coverslide resulting in an evenly dispersed distribution of particles.

## Additional Information

**How to cite this article**: Duarte, A. S. *et al*. Wide-Field Detected Fourier Transform CARS Microscopy. *Sci. Rep.* **6**, 37516; doi: 10.1038/srep37516 (2016).

**Publisher's note:** Springer Nature remains neutral with regard to jurisdictional claims in published maps and institutional affiliations.
